# Methane-Rich Saline Ameliorates Sepsis-Induced Acute Kidney Injury through Anti-Inflammation, Antioxidative, and Antiapoptosis Effects by Regulating Endoplasmic Reticulum Stress

**DOI:** 10.1155/2018/4756846

**Published:** 2018-11-15

**Authors:** Yifan Jia, Zeyu Li, Yang Feng, Ruixia Cui, Yanyan Dong, Xing Zhang, Xiaohong Xiang, Kai Qu, Chang Liu, Jingyao Zhang

**Affiliations:** ^1^Department of Hepatobiliary Surgery, The First Affiliated Hospital of Xi'an Jiaotong University, Xi'an Shaanxi 710061, China; ^2^Department of Immunology, Shaanxi University of Chinese Medicine, Xianyang Shaanxi 712046, China; ^3^Department of ICU, The First Affiliated Hospital of Xi'an Jiaotong University, Xi'an Shaanxi 710061, China; ^4^Department of SICU, The First Affiliated Hospital of Xi'an Jiaotong University, Xi'an Shaanxi 710061, China

## Abstract

Sepsis-induced acute kidney injury (AKI) is a severe complication of sepsis and an important cause of mortality in septic patients. Previous investigations showed that methane had protective properties against different diseases in animal models. This study is aimed at investigating whether methane-rich saline (MRS) has a protective effect against sepsis-induced AKI. Sepsis was induced in wild-type C57BL/6 mice by cecal ligation and puncture (CLP), and the mice were divided into three groups: a sham control group (sham), a surgery group with saline intraperitoneal injection (i.p.) treatment (CLP + NS), and a surgery group with MRS i.p. treatment (CLP + MRS). 24 h after the establishment of the sepsis, the blood and kidney tissues of mice in all groups were collected. According to the serum levels of blood urea nitrogen (BUN) and creatinine (CRE) and a histologic analysis, which included hematoxylin-eosin (H&E) staining and periodic acid-Schiff (PAS) staining, MRS treatment protected renal function and tissues from acute injury. Additionally, MRS treatment significantly ameliorated apoptosis, based on the levels of apoptosis-related protein makers, including cleaved caspase-3 and cleaved PARP, and the levels of Bcl-2/Bax expression and TUNEL staining. In addition, the endoplasmic reticulum (ER) stress-related glucose-regulated protein 78 (GRP78)/activating transcription factor 4 (ATF4)/C/EBP homologous protein (CHOP)/caspase-12 apoptosis signaling pathway was significantly suppressed in the CLP + MRS group. The levels of inflammation and oxidative stress were also reduced after MRS treatment. These results showed that MRS has the potential to ameliorate sepsis-induced acute kidney injury through its anti-inflammatory, antioxidative, and antiapoptosis properties.

## 1. Introduction

Sepsis-induced acute kidney injury (AKI) is a severe complication of sepsis and a leading cause of mortality in intensive care unit (ICU) patients. Among critically ill patients, the morbidity of acute renal injury can be up to 70%, and approximately 5% of these patients progress to acute renal failure during their hospital stays. The overall ICU mortality rate of acute renal failure is approximately 50%, and 15% of survivors continued to rely on renal replacement therapy (RRT) after they were discharged. The leading cause of acute kidney injury is sepsis, which contributes to the 50% of the incidence [1–4]. Despite advances in clinical treatment and intensive care, there is currently no specific therapy for septic AKI; however, the early onset of RRT may reduce mortality [5]. Septic AKI is the result of a series of complex interactions between vascular endothelial cell dysfunction, subsequent inflammation, and tubular cell damage [6]. Additionally, recent research has suggested that apoptosis and immune suppression, especially the apoptosis of tubular cells, may be involved in the pathological process of septic AKI, which is quite different from the other types of AKI [7].

During the sepsis process, the accumulation of unfolded protein in endoplasmic reticulum (ER), which is called ER stress, triggers an evolutionarily conserved unfolded protein response (UPR) to reestablish cellular homeostasis. Three transmembrane proteins, including inositol-requiring enzyme 1 (IRE1*α*), PKR-like ER kinase (PERK), and activating transcription factor 6 (ATF6), are initially activated and accelerate the unfolded protein response by activating downstream signaling pathways. However, prolonged UPR can lead to an excessive ER stress and result in C/EBP homologous protein- (CHOP-) mediated apoptosis [8]. During sepsis-induced acute kidney injury, ER stress also plays an important role [9, 10].

Methane, the simplest organic compound, can be detected in 30%–50% of healthy adults around the world and has been deemed to have little physiological activity for decades. However, many recent studies have discovered that methane has several important biological effects that can protect cells and organs from inflammation, oxidative stress, and apoptosis [11–14]. It is notable that methane could play a protective role in the cellular apoptosis process. Methane could remarkably attenuate the expression of the proapoptotic protein Bax and increase the expression of Bcl-2, which resulted in the downregulation of caspase-3 and other downstream caspases, thus suppressing the activation of the mitochondrial apoptotic pathway [15]. Based on these protective effects, methane has been considered a possible therapeutic agent for several diseases, including acute lung injury, autoimmune hepatitis, and spinal cord injury [13, 16, 17]. However, there has been little investigation regarding whether methane can protect the kidney from septic AKI. Accordingly, we hypothesize that methane has a protective effect against septic AKI and aim to prove it using a CLP-induced sepsis model. We also examined whether ER stress played a key role in the mechanism underlying this effect and provided some suggestions for the use of methane in clinical practice.

## 2. Materials and Methods

### 2.1. Animals

Healthy female wild-type C57BL/6 mice (6–7 weeks old, weighing 20–25 g) were obtained from the Animal Feeding Center of Xi'an Jiaotong University Health Science Center. All the mice were maintained at a constant temperature of 25°C and a humidity of 50% and fed with a standard diet with open access to tap water under a 12 : 12 day/night cycle for 7 days before the experiments. The animal experiment was performed in accordance with the Guide of Laboratory Animal Care and Use from the United States National Institution of Health and was approved and supervised by the Institutional Animal Care and Use Committee of the Ethics Committee of Xi'an Jiaotong University Health Science Center, China.

### 2.2. Animal Model of CLP

The sepsis model was established as previously described [18]. Mice were anesthetized by 50 mg/kg pentobarbital sodium (i.p.), and laparotomy was performed to isolate the cecum of mice along the midline of the abdomen, ligating the 1/3 cecal tip with a 4-0 silk suture. The cecum was separated with forceps and punctured twice, and one column of fecal material was squeezed out. The cecum was returned to its original location, and the abdomen closed in layers with a 4-0 silk suture. The mice were put back in their cages until they completely recovered. The sham operation was performed by only incising the abdomen, separating and exposing the cecum for 5 minutes, then closing it in layers.

### 2.3. Experimental Groups and Drug Treatment

Mice were randomly divided into the three groups: (1) sham group (*n* = 6): the C57BL/6 mice received a sham laparotomy operation. (2) Normal saline group (*n* = 12): the C57BL/6 mice underwent a laparotomy operation with CLP; meanwhile, the normal saline was administered at 10 mL/kg dosage every 4 hours as a control (CLP + NS group). (3) Methane-rich saline group (*n* = 12): the C57BL/6 mice received a laparotomy operation with CLP and were given an intraperitoneal injection (i.p.) of methane-rich saline (MRS) at 10 mL/kg (CLP + MRS group) every 4 hours after a successful sepsis model establishment [14]. 24 hours after the surgery, all animals were euthanized; kidney and blood samples were collected for quantification of biochemical analysis.

### 2.4. Methane-Rich Saline Production

The production method of the MRS was described by Ye et al. [19]. Briefly, normal saline was saturated with pure methane (>99.999%) in a high-pressure vessel (Wuhan Newradar Special Gas Co. Ltd., Wuhan, China) under 0.4 MPa for 4 h, stored at 4°C, and freshly prepared 24 h before administration to the animals to ensure the methane concentration. The concentration of the methane was detected as previous described [20].

### 2.5. Renal Function Analysis

Blood samples (*n* = 6) were collected from the eyeballs of the mice and centrifuged at 4°C for 15 min at 1500 × *g* in tubes. Renal functions were evaluated by BUN and CRE levels, which were detected by a Hitachi 7600-20 automatic biochemical analyzer (Clinical Laboratory of the First Affiliated Hospital of Xi'an Jiaotong University, Xi'an, China).

### 2.6. Histopathological Examination

Kidney tissue samples (*n* = 6) were gathered 24 h after surgery and fixed in 10% formalin solution. Serial 5 *μ*m sections were stained with hematoxylin and eosin (H&E). The histologic changes and the quantity of infiltrated neutrophils were assessed in a double-blinded way to randomly select five different fields in each sample through the light microscope. Then, the samples were embedded in paraffin and sectioned into 5 *μ*m thick sections and stained with periodic acid-Schiff (PAS). Histological changes in the cortex and in the outer stripe of the outer medulla (OSOM) were assessed by quantitative measures of tissue damage by a blind observation. Tubular damage was defined as tubular epithelial swelling, loss of brush border, vacuolar degeneration, necrotic tubules, cast formation, and desquamation. The degree of kidney damage was estimated at ×200 magnification, using five randomly selected fields for each animal, by the following criteria: 0, normal; 1, area of damage < 25% of tubules; 2, damage involving 25–50% of tubules; 3, damage involving 50–75% of tubules; and 4, damage involving 75–100% of tubules [21].

### 2.7. Cytokine Detection

Kidney tissues from different groups (*n* = 6) were homogenized with PBS on ice and then centrifuged at 4°C for 40 min at 12,000 rpm. Subsequently, the supernatants were collected to perform ELISA. We used commercially available ELISA kits to detect the levels of inflammatory cytokines, including TNF-*α*, IL-6, and IL-1*β* (Jiancheng Bioengineering Institute, Nanjing, China), in kidney tissue according to the manufacturer's recommendation.

### 2.8. Reactive Oxidative Stress Activity Assay

The kidney tissue (*n* = 6) homogenate produced was previously described to measure the oxidative stress indexes. The level of malondialdehyde (MDA) in the tissue was taken as the level of lipid oxidation in the tissue, while the superoxide dismutase (SOD) was the index of the tissue antioxidant. The levels of MDA and the activities of SOD in kidney tissues were detected by commercial biochemical kits (Jiancheng Bioengineering Institute, Nanjing, China) following the manufacturer's instructions.

### 2.9. Detection of ROS Activation

Tissue samples (*n* = 6) were fixed with 10% formaldehyde and dehydrated using 30% sucrose solution. The sections (4 *μ*m thick) were incubated with dihydroethidium (DHE; 10 *μ*M) (Vigorous Biotechnology Co. Ltd., Beijing, China). After 60 min in the dark, the specimens were washed with adequate volumes of PBS. DHE oxidized by ROS in the cells could show red emission.

### 2.10. Western Blot Assay

The protein expression in kidney tissue (*n* = 6) was detected by Western blot. In brief, RIPA lysis buffer was used to extract the total protein and nucleoprotein at 14000*g* for 15 min at 4°C. After the protein concentration was determined, the lysates were separated using sodium dodecyl sulfate-polyacrylamide gel electrophoresis (SDS-PAGE). The proteins were transferred onto polyvinylidene difluoride (PVDF) membranes. The resulting blots were blocked with 8% skim milk and incubated with an anti-GRP78 antibody (1 : 5000; Proteintech, China), an anti-ATF4 antibody (1 : 1000; Proteintech, China), anti-caspase-12 antibodies (1 : 1000; Cell Signaling Technology (CST), USA), anti-IL-1*β* antibodies (1 : 1000; Abcam, USA), anti-TNF-*α* (1 : 500; Abcam, USA), anti-Bcl2 antibodies (1 : 1000; Biosis, China), anti-Bax antibodies (1 : 2000; Proteintech, China), anti-cleaved-caspase-3 antibodies (1 : 1000; Cell Signaling Technology (CST), USA), anti-PARP antibodies (1 : 1000; Cell Signaling Technology (CST), USA), and anti-*β*-actin antibodies (1 : 10000; Santa Cruz Biotechnology, USA) overnight at 4°C. Subsequently, the blots were washed three times with PBS and incubated with anti-rabbit and anti-mouse horseradish peroxidase-conjugated secondary antibodies (1 : 10000; Abmart, China) for 1 h at 37°C. The proteins were detected with the chemiluminescence (ECL) system. The expressions of proteins were normalized to *β*-actin as a reference.

### 2.11. Immunohistochemical Analysis

Twenty-four hours after the CLP operation, the kidney tissues (*n* = 6) were gathered and processed for immunohistochemistry to estimate the activation of CHOP and IL-1*β*. Briefly, the kidney tissues were fixed into 4% paraformaldehyde and then embedded in paraffin and cut into 4 *μ*m thick sections. The sections were deparaffinized with xylene and rehydrated with serial gradient ethanol. After incubation with 3% hydrogen peroxide for 15 min to block endogenous peroxidase activity, the sections were blocked with goat serum and then incubated with primary antibodies against CHOP (1 : 500; Bioss, China) and IL-1*β* (1 : 150; Proteintech, China) overnight at 4°C. After those procedures, the sections were washed with PBS three times and incubated with biotinylated secondary antibodies at room temperature for 40 min. Finally, the sections were incubated with diaminobenzidine tetrahydrochloride (DAB), counterstained with hematoxylin, and mounted for microscopic examination. The images were selected at 200× magnification, and five fields were captured randomly.

### 2.12. Detection of the Apoptosis Rate by TUNEL Staining

The apoptosis rate of the kidney tissue (*n* = 6) was detected by TdT-mediated dUTP nick-end labeling (TUNEL) staining 24 h after sepsis. The experiment was carried out strictly according to the instructions of the TUNEL kit (Roche Molecular Biochemicals, Indianapolis, IN, USA). The images were selected at 200× magnification, and five fields were captured randomly.

### 2.13. Statistical Analysis

The measurement data were described as the mean ± standard deviation (SD). All statistical analyses were performed by the SPSS 18.0 software (SPSS Inc., Chicago, USA). Student's *t*-test or one-way analysis of variance (ANOVA) was used for the comparison among the groups. The figure was made by GraphPad (version 7) Prism software (GraphPad Software, La Jolla, CA). All tests were two sided, and significance was accepted at *P* < 0.05.

## 3. Results

### 3.1. Methane-Rich Saline Protected Kidney Functions in CLP Mice

**Figure 1:**
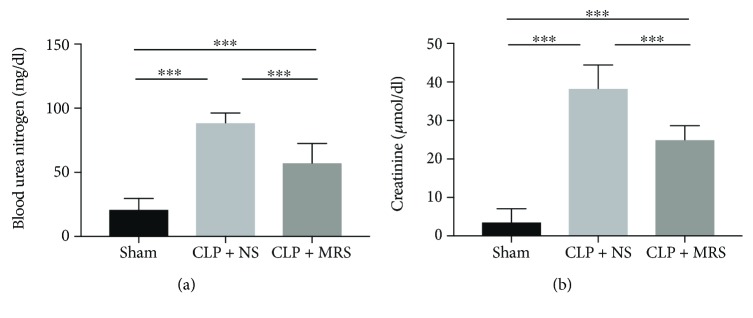
Methane-rich saline protected kidney function in CLP mice. (a) The blood urea nitrogen level in mouse serum. (b) The creatinine level in mice serum. ^∗^*P* < 0.05, ^∗∗^*P* < 0.01, and ^∗∗∗^*P* < 0.001.

First, we investigated whether MRS can improve kidney function during the sepsis process. Blood urea nitrogen (BUN) and serum creatinine (CRE) are the products of protein metabolism and are used as indicators of kidney function. Elevated levels of BUN and CRE were observed after the establishment of the CLP model, confirming that the sepsis model was successfully constructed (Figure 1). However, in the CLP + MRS group, the levels of BUN and CRE were reduced dramatically compared to those of the CLP + NS group (*P* < 0.05).

### 3.2. Methane-Rich Saline Attenuated Histopathological Damage in the Kidneys of CLP Mice

**Figure 2:**
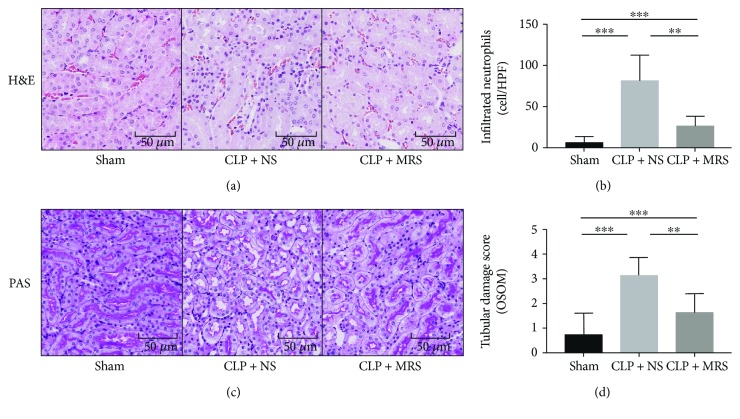
Methane-rich saline attenuated kidney histopathological damage in CLP mice. (a) H&E staining was performed on kidney tissue slices 24 h after CLP. (b) The infiltrated neutrophil granulocytes were counted. (c) PAS staining was performed on kidney tissue slices 24 h after CLP. (d) The tubular damage scores of kidney tissue were calculated. Representative sections; original magnification, 200x. ^∗^*P* < 0.05, ^∗∗^*P* < 0.01, and ^∗∗∗^*P* < 0.001.

We then attempted to confirm the histopathological damage among the different groups. The H&E staining was performed on kidney tissue slices. There was little histopathological damage observed in the sham group. Widespread degeneration and necrosis were observed in tubular epithelial cells in the CLP + NS group. However, there was slight damage, including tubular epithelial swelling and brush border injury, in the CLP + MRS group (Figure 2(a)). We calculated the number of infiltrated neutrophils (Figure 2(b)) and found that the MRS treatment could greatly ameliorate the severity of kidney destruction. Moreover, the PAS staining and the tubular damage score also showed the same protective effect of MRS on kidney histology (Figures 2(c) and 2(d)).

### 3.3. Anti-Inflammation Effects of Methane-Rich Saline on Septic AKI

**Figure 3:**
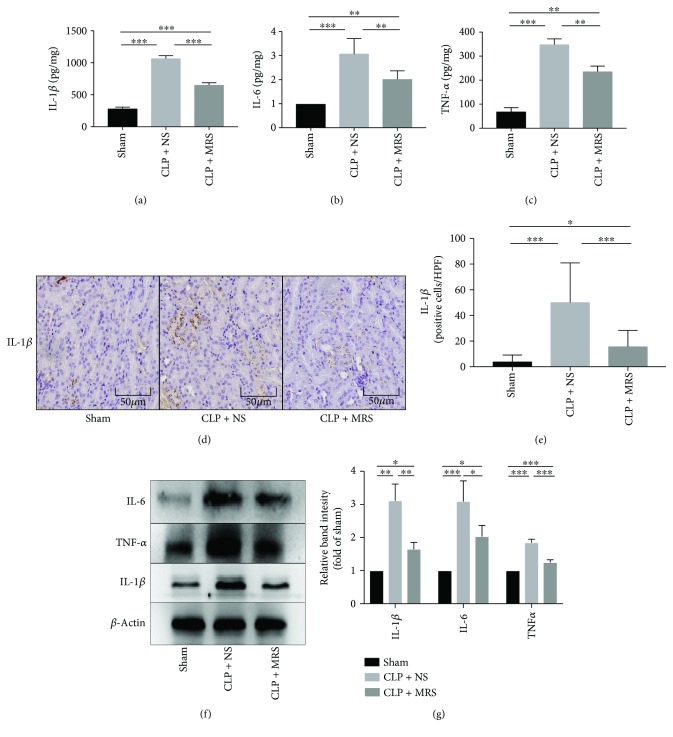
Methane-rich saline suppressed inflammation response in kidney tissue after CLP. The levels of (a) IL-1*β*, (b) IL-6, and (c) TNF-*α* in kidney tissue were assessed by ELISA. (d) The expression of IL-1*β* was detected by immunohistochemistry. (e) The quantity of IL-1*β*-positive cells was counted in a high-power field. (f) The kidney protein expression levels of IL-1*β*, IL-6, and TNF-*α* were detected by Western blot. (g) The relative band intensity (fold of the sham group) were shown. Representative sections; original magnification, 200x. ^∗^*P* < 0.05, ^∗∗^*P* < 0.01, and ^∗∗∗^*P* < 0.001.

An unresolved inflammation response plays a crucial role in sepsis. As previous research has reported, MRS can attenuate organism damage by suppressing the overactivation of the inflammatory response. We detected the levels of proinflammatory cytokines in kidney tissues from the different groups, including IL-1*β*, IL-6, and TNF-*α* by ELISA (Figures 3(a), 3(b), and 3(c)) and Western blot (Figures 3(f) and 3(g)). According to the results, the levels of IL-1*β*, IL-6, and TNF-*α* were all increased after CLP surgery. Compared with the CLP + NS group, the levels of the proinflammatory cytokines were reduced in the CLP + MRS group, which confirmed the anti-inflammation effect of MRS. Then, we performed an immunohistochemical analysis of IL-1*β* in kidney tissues from the different groups (Figures 3(d) and 3(e)). The results of the immunohistochemical analysis demonstrated similar results to those from the ELISA and the Western blot. According to the results, the sham group had few IL-1*β*-positive cells. However, the CLP + NS group had a more obvious emergence of IL-1*β*-positive cells than the CLP + MRS group. All these results showed that MRS has a very strong inflammatory suppression effect.

### 3.4. Antioxidative Effects of Methane-Rich Saline on Septic AKI

**Figure 4:**
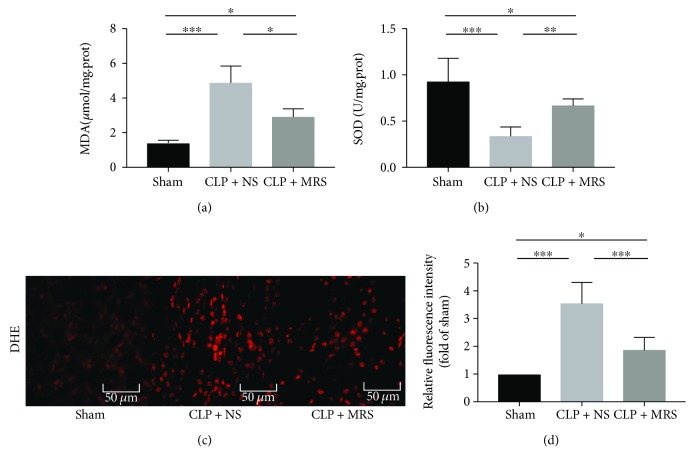
Methane-rich saline attenuated oxidative stress in kidney tissue after CLP. The levels of (a) MDA and (b) SOD in kidney tissue were assessed. (c) The DHE fluorescent probe was performed on kidney tissue slices. (d) The relative fluorescence intensities (fold of sham) of ROS were shown. Representative sections; original magnification, 200x. ^∗^*P* < 0.05, ^∗∗^*P* < 0.01, and ^∗∗∗^*P* < 0.001.

The activation of oxidative stress, including reactive oxygen species (ROS), also occurs during sepsis and induces organ and tissue damage. Oxidative stress levels above a certain threshold can trigger an ER stress overload and induce the activation of CHOP-mediated apoptosis. Simultaneously, excessive ER stress can also lead to harmful oxidative stress. We detected the serum MDA and SOD levels 24 h after the CLP operation (Figures 4(a) and 4(b)). The results showed that the CLP + NS group had the highest MDA level among the three groups, and the level in the CLP + MRS group was significantly reduced compared to that in the CLP + NS group. Moreover, the results indicated that SOD activity of the CLP + MRS group was greater than that of the CLP + NS group. To reveal the different levels of oxidative stress in a visual way, we measured the ROS levels in kidney tissues by DHE, and different levels of fluorescence intensity revealed different levels of ROS activity (Figures 4(c) and 4(d)). The results demonstrated that the ROS activity in the CLP + MRS group was attenuated when compared with that of the CLP + NS group, as the DHE fluorescence intensity was decreased in the CLP + MRS group compared with the CLP + NS group. According to these results, MRS has an antioxidative effect on the kidney during sepsis.

### 3.5. Antiapoptosis Effects of Methane-Rich Saline on Septic AKI

**Figure 5:**
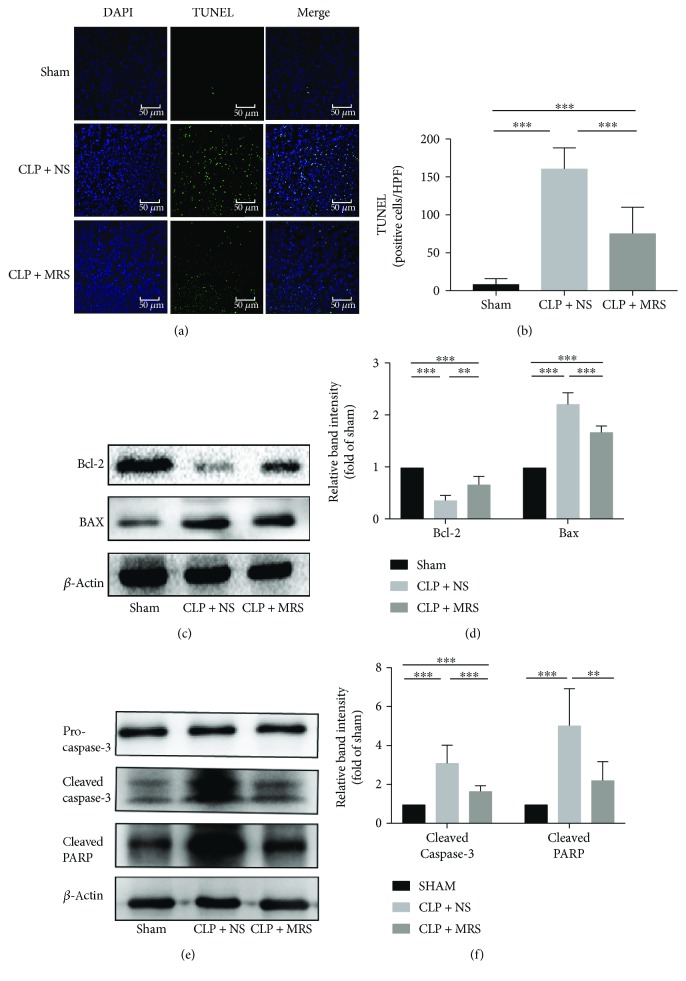
Antiapoptosis effects of methane-rich saline on septic AKI. (a) TUNEL assay was performed on kidney tissue slices. (b) The quantity of TUNEL-positive cells was counted in a high-power field. (c) The kidney protein expression levels of Bcl-2 and Bax were detected by Western blot, and the relative band intensities (fold of the sham group) were shown in (d). (e) The protein levels of cleaved caspase-3 and cleaved PARP were detected, and the relative band intensities (fold of the sham group) were shown in (f). ^∗^*P* < 0.05 versus CLP + NS group; representative sections; original magnification, 200x. ^∗^*P* < 0.05, ^∗∗^*P* < 0.01, and ^∗∗∗^*P* < 0.001.

As an important link in sepsis-related acute kidney injury, apoptosis of the cells in the kidney, especially tubular endothelial cells, could lead to major damage in the septic kidney. We examined apoptotic cells using a TUNEL system (Figures 5(a) and 5(b)). Two researchers counted the number of TUNEL-positive cells. A significant increase was observed in the number of TUNEL-positive tubular cells in the CLP + NS group compared with the sham group. However, the CLP + MRS group had a greatly reduced number of TUNEL-positive cells compared with the CLP + NS group. In agreement with the TUNEL assay results, the CLP operation also led to a significant augmentation of Bax and a decline in Bcl-2 expression in comparison with the sham group, and MRS treatment reduced the level of Bax and increased the level of Bcl-2 (Figures 5(c) and 5(d)). In addition, MRS could also attenuate the high expression of cleaved caspase-3 and PARP induced by CLP (Figures 5(e) and 5(f)). These results revealed that there was significant apoptotic activity in the cells of the kidney during sepsis and that MRS treatment could dramatically reduce apoptosis.

### 3.6. Methane-Rich Saline Ameliorated the ER Stress-Related Apoptosis Process in Septic AKI

**Figure 6:**
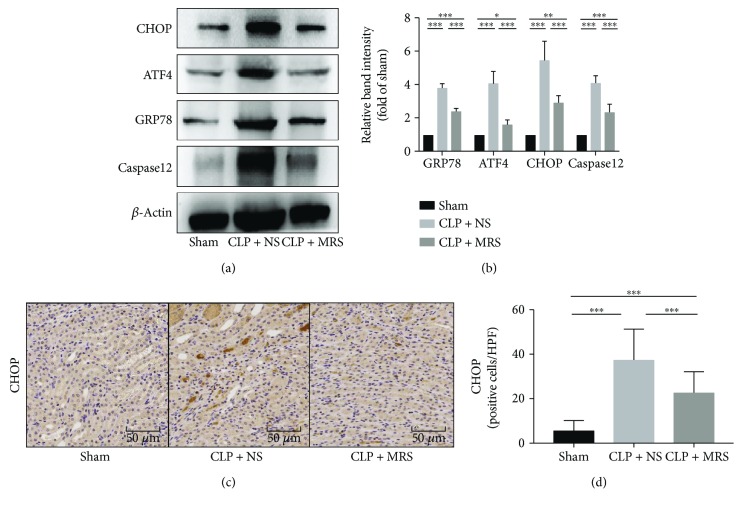
Methane-rich saline ameliorated the ER stress-related apoptosis process in septic AKI. (a) The protein levels of ER stress-related apoptosis signaling components (GRP78/ATF4/CHOP/caspase-12) were detected by Western blot, and the relative band intensities (fold of the sham group) were shown in (b). CHOP was detected by immunohistochemistry, and the quantity of CHOP-positive cells was counted in a high-power field (c). Representative sections; original magnification, 200x. ^∗^*P* < 0.05, ^∗∗^*P* < 0.01, and ^∗∗∗^*P* < 0.001.

ER stress played an important role in sepsis-induced AKI; therefore, we tested the expression levels of the ER stress proteins and related components of apoptosis signaling pathways. We examined the levels of GRP78/ATF4/CHOP/caspase-12 by Western blot (Figures 6(a) and 6(b)). An increasing trend could be identified in the CLP + NS group, which showed the activation of ER stress-related apoptosis. However, there was an obvious decreasing trend in the CLP + MRS group when compared to the CLP + NS group. Moreover, we also assessed the level of CHOP using immunohistochemical analysis (Figures 6(c) and 6(d)). As the immunohistochemical analysis images show, the methane-rich saline effectively reduced the number of the CHOP-positive cells. Hence, we suggest that methane has a downregulating effect on ER stress-related apoptosis.

## 4. Discussion

Sepsis-related AKI is a severe complication following sepsis and leads to high mortality [22]. Although many studies have been performed on sepsis-related AKI, they have mostly focused on the pathophysiological mechanisms and risk factors. However, the treatment of sepsis-related AKI is still limited, and thus, the mortality is still a challenging problem in clinical practice.

Methane, the simplest alkane and likely the most abundant organic compound on earth, has been used as gas fuel by people for hundreds of years. Recently, scientists have focused on the clinical properties of methane and have suggested that methane can influence several pathological processes, including inflammation, oxidation, and apoptosis in different diseases [12–14]. However, the mechanisms behind these effects could be complex and have yet to be investigated. Some researchers, when examining a carbon tetrachloride-induced liver injury, have suggested that methane exhibits an anti-inflammatory effect through the activation of the PI3K-AKT-GSK-3*β* pathway, which induces IL-10 expression and produces anti-inflammatory effects via the NF-*κ*B and MAPK pathways. In addition, Nrf2 and its downstream pathway have been suggested to be involved in the anti-inflammation and antioxidant effects in spinal cord ischemia-reperfusion injury [14].

There has been little research regarding the protective effects of methane on serious sepsis-induced acute kidney injury. Some previous studies have demonstrated that inflammation, oxidative stress, and apoptosis, especially the apoptosis of tubular endothelial cells, play important roles in sepsis-related AKI [23]. This study is aimed at revealing the protective effects of methane on sepsis-related AKI and at determining the underlying mechanisms.

We created a sepsis mouse model using a CLP operation and gathered the blood and kidney tissue samples after 24 hours. First, we evaluated the renal functions of all groups and the levels of BUN and CRE after CLP to confirm the establishment of a sepsis model. The MRS treatment demonstrated protective properties for kidney function during serious sepsis, and the results were confirmed using histopathological damage analysis. We next attempted to investigate which parts of pathophysiological process had been influenced by MRS. We assessed the levels of the inflammatory response and oxidative stress among the different groups and found a significantly decreasing trend in the CLP + MRS group, which suggested that MRS treatment has strong anti-inflammation and antioxidative effects. As a crucial part of sepsis-related AKI, we examined cell apoptosis in renal tissues, especially the expression of cleaved caspase-3, PARP, and Bcl-2/Bax, and demonstrated an antiapoptosis effect of MRS treatment. We revealed a protective effect for MRS treatment against sepsis-related AKI that was mediated through anti-inflammation, antioxidative, and antiapoptosis effects.

Apoptosis is a process of programmed cell death that occurs in multicellular organisms and is important for homeostasis in multicellular life forms. The apoptosis of mammalian cells can be triggered by various stimuli via the intrinsic pathways, mitochondrial pathway and ER pathway.

Endoplasmic reticulum (ER) stress is a stress condition in which unfolded or misfolded proteins amass in the endoplasmic reticulum. While a mild ER stress-induced UPR signaling pathway can be considered a maintenance mechanism, chronically prolonged ER stress can deteriorate cellular functions and transform the adaptation programmed into CHOP-mediated apoptosis, activating the caspase-12 and Bcl-2/Bax system to clean irreversibly injured cells [24, 25]. We further investigated the level of ER stress-related apoptosis and discovered that MRS treatment could attenuate ER stress-related apoptosis, which is consistent with the previous results demonstrating the antiapoptosis effects of MRS. The levels of CHOP and caspase-12 were both reduced in the CLP + MRS group when compared with the CLP + NS group. In addition, the levels of cleaved caspase-3 and PARP as well as Bax were suppressed in the CLP + MRS group.

The exact molecular mechanisms of methane have not been clarified. Several scholars have proposed different hypotheses to explain the protective functions of methane, including the regulation of embedded proteins in the cell membrane, an interaction with a specific receptor, or ion channel kinetics [26, 27]. According to our research, we found that the reduction of ER stress may be involved in the protective mechanism of methane and could inhibit cell apoptosis directly. Therefore, we hypothesize that methane might block some signaling pathway triggers of ER stress and raise the threshold of the ER stress-related apoptosis, so that methane could also stop the downstream inflammation and oxidative signals. Many studies have confirmed that ER stress is involved in several pathological processes and contributes to tissue damage. As a potential ER stress-related apoptosis inhibitor and a promising medicinal treatment for sepsis-induced AKI, MRS could be applied as a valid intervention during the early stages of sepsis and for establishing a protective effect on the kidney and other organs. In conclusion, this study demonstrated that methane treatment reduced the inflammatory response, oxidative stress, and apoptosis during sepsis-induced AKI, suggesting that the reduction of ER stress may be the underlying protective mechanism.

## Data Availability

The histological examination, biochemical index detection, results of IHC and TUNEL staining, and WB data used to support the findings of this study are included within the article.

## References

[1] Ricci Z., Cruz D., Ronco C. (2008). The RIFLE criteria and mortality in acute kidney injury: a systematic review. *Kidney International*.

[2] Uchino S., Kellum J. A., Bellomo R. (2005). Acute renal failure in critically ill patients: a multinational, multicenter study. *JAMA*.

[3] Mehta R. L., Pascual M. T., Soroko S. (2004). Spectrum of acute renal failure in the intensive care unit: the PICARD experience. *Kidney International*.

[4] Silvester W., Bellomo R., Cole L. (2001). Epidemiology, management, and outcome of severe acute renal failure of critical illness in Australia. *Critical Care Medicine*.

[5] Kresse S., Schlee H., Deuber H. J., Koall W., Osten B. (1999). Influence of renal replacement therapy on outcome of patients with acute renal failure. *Kidney International Supplements*.

[6] Jacobs R., Honore P. M., Joannes-Boyau O. (2011). Septic acute kidney injury: the culprit is inflammatory apoptosis rather than ischemic necrosis. *Blood Purification*.

[7] Lee S. Y., Lee Y. S., Choi H. M. (2012). Distinct pathophysiologic mechanisms of septic acute kidney injury: role of immune suppression and renal tubular cell apoptosis in murine model of septic acute kidney injury. *Critical Care Medicine*.

[8] Hetz C. (2012). The unfolded protein response: controlling cell fate decisions under ER stress and beyond. *Nature Reviews Molecular Cell Biology*.

[9] Teng J., Liu M., Su Y. (2018). Down-regulation of GRP78 alleviates lipopolysaccharide-induced acute kidney injury. *International Urology and Nephrology*.

[10] Esposito V., Grosjean F., Tan J. (2013). CHOP deficiency results in elevated lipopolysaccharide-induced inflammation and kidney injury. *American Journal of Physiology-Renal Physiology*.

[11] Jia Y., Li Z., Liu C., Zhang J. (2018). Methane medicine: a rising star gas with powerful anti-inflammation, antioxidant, and antiapoptosis properties. *Oxidative Medicine and Cellular Longevity*.

[12] Yao Y., Wang L., Jin P. (2017). Methane alleviates carbon tetrachloride induced liver injury in mice: anti-inflammatory action demonstrated by increased PI3K/Akt/GSK-3β-mediated IL-10 expression. *Journal of Molecular Histology*.

[13] Sun A., Wang W., Ye X. (2017). Protective effects of methane-rich saline on rats with lipopolysaccharide-induced acute lung injury. *Oxidative Medicine and Cellular Longevity*.

[14] Wang L., Yao Y., He R. (2017). Methane ameliorates spinal cord ischemia-reperfusion injury in rats: antioxidant, anti-inflammatory and anti-apoptotic activity mediated by Nrf2 activation. *Free Radical Biology & Medicine*.

[15] Liu L., Sun Q., Wang R. (2016). Methane attenuates retinal ischemia/reperfusion injury via anti-oxidative and anti-apoptotic pathways. *Brain Research*.

[16] He R., Wang L., Zhu J. (2016). Methane-rich saline protects against concanavalin A-induced autoimmune hepatitis in mice through anti-inflammatory and anti-oxidative pathways. *Biochemical and Biophysical Research Communications*.

[17] Wang W., Huang X., Li J. (2017). Methane suppresses microglial activation related to oxidative, inflammatory, and apoptotic injury during spinal cord injury in rats. *Oxidative Medicine and Cellular Longevity*.

[18] Zhang J., Bi J., Liu S. (2017). 5-HT drives mortality in sepsis induced by cecal ligation and puncture in mice. *Mediators of Inflammation*.

[19] Ye Z., Chen O., Zhang R. (2015). Methane attenuates hepatic ischemia/reperfusion injury in rats through antiapoptotic, anti-inflammatory, and antioxidative actions. *Shock*.

[20] Ohsawa I., Ishikawa M., Takahashi K. (2007). Hydrogen acts as a therapeutic antioxidant by selectively reducing cytotoxic oxygen radicals. *Nature Medicine*.

[21] Miyaji T., Hu X., Yuen P. S. T. (2003). Ethyl pyruvate decreases sepsis-induced acute renal failure and multiple organ damage in aged mice. *Kidney International*.

[22] Brivet F. G., Kleinknecht D. J., Loirat P., Landais P. J. M. (1996). Acute renal failure in intensive care units--causes, outcome, and prognostic factors of hospital mortality; a prospective, multicenter study. French study group on acute renal failure. *Critical Care Medicine*.

[23] Zarbock A., Gomez H., Kellum J. A. (2014). Sepsis-induced acute kidney injury revisited: pathophysiology, prevention and future therapies. *Current Opinion in Critical Care*.

[24] Szegezdi E., Logue S. E., Gorman A. M., Samali A. (2006). Mediators of endoplasmic reticulum stress-induced apoptosis. *EMBO Reports*.

[25] Rutkowski D. T., Arnold S. M., Miller C. N. (2006). Adaptation to ER stress is mediated by differential stabilities of pro-survival and pro-apoptotic mRNAs and proteins. *PLoS Biology*.

[26] Boros M., Ghyczy M., Érces D. (2012). The anti-inflammatory effects of methane^∗^. *Critical Care Medicine*.

[27] Kai T., Jones K. A., Warner D. O. (1998). Halothane attenuates calcium sensitization in airway smooth muscle by inhibiting G-proteins. *Anesthesiology*.

